# Atrial time and voltage dispersion are both needed to predict new-onset atrial fibrillation in ischemic stroke patients

**DOI:** 10.1186/s12872-017-0631-1

**Published:** 2017-07-24

**Authors:** Daniel Cortez, Maria Baturova, Arne Lindgren, Jonas Carlson, Yuri V. Shubik, Bertil Olsson, Pyotr G. Platonov

**Affiliations:** 1 Department of Cardiology, Clinical Sciences, Lund University, Lund, Sweden; 2 Electrophysiology Department, Penn State Milton S. Hershey Medical Center, Hershey, USA; 3 St. Petersburg University Clinic, St. Petersburg, Russia; 4 Cardiology Research, Clinical and Educational Center, St. Petersburg State University, St. Petersburg, Russia; 5 Department of Neurology and Rehabilitation Medicine, Skane University Hospital, Lund, Sweden; 6 Department of Clinical Sciences Lund, Neurology, Lund University, Lund, Sweden; 7 Arrhythmia Clinic, Skåne University Hospital, Lund, Sweden

**Keywords:** Atrial fibrillation, Ischemic stroke, P-wave vector magnitude, P-wave duration

## Abstract

**Background:**

Atrial fibrillation (AF) is a known risk factor for ischemic stroke. Electrocardiographic predictors of AF in population studies such as the Framingham Heart Study, as well as in hypertensive patients have demonstrated a predictive value of the P-wave duration for development of AF. QRS vector magnitude has had a predictive value in ventricular arrhythmia development. We aimed to assess the value of the three-dimensional P-wave vector magnitude and its relationship to P-wave duration for prediction of new-onset AF after ischemic stroke.

**Methods:**

First-ever ischemic stroke patients without AF at inclusion in the Lund Stroke Register were included. Measurements of P wave duration (Pd), QRS duration, corrected QT interval, and PQ interval were performed automatically using the University of Glasgow 12-lead ECG analysis algorithm. The P-wave vector magnitude (Pvm) was calculated automatically as the square root of the sum of the squared P-wave magnitudes in leads V6, II and one half of the P-wave amplitude in V2 ($\sqrt{PV{6}^2+{PII}^2+{\left({0.5}^{\ast }PV2\right)}^2}$), based on the P-wave magnitude (Pvm) as defined by the visually transformed Kors’ Quasi-orthogonal method.

**Results:**

The median age was 73 (IQR 63–80) years at stroke onset (135 males, 92 females). Multivariate predictors of new-onset atrial fibrillation included age > 65 years, hypertension, and Pd/Pvm. A cut-off value of 870 ms/mV gave sensitivity, specificity, positive and negative predictive values of 51, 79, 30 and 87%, respectively. The Pd/Pvm was the only ECG predictor of AF with a significant multivariate hazard ratio of 2.02 (95% CI 1.18 to 3.46, *p* = 0.010).

**Conclusion:**

P-wave dispersion as measured by the Pd/Pvm was the only ECG parameter measured which independently predicted subsequent AF identification in a cohort of stroke patients. Further prospective studies in larger cohorts are needed to validate its clinical usefulness.

## Background

Atrial fibrillation (AF) is a known risk factor for ischemic stroke [1]. A high prevalence of AF is noted in ischemic stroke patients [1]. The impact of ischemic stroke on the risk of subsequent development of AF is only beginning to become clear [2, 3]. Information on development of new AF in ischemic stroke patients using ECG monitoring has been seldom reported until recently [4–6]. Clinical cardiovascular risk scoring tools such as the CHADS_2_ and CHA_2_DS_2_-VASc have demonstrated association with development of first-ever AF during 2-year and 10-year follow-up time frames in recent studies [7–9].

Electrocardiographic predictors of AF in populations such as the Framingham Heart Study, as well as in hypertensive patients have demonstrated a predictive value of the p-wave duration for development of AF [10, 11]. This parameter, however, was not predictive in ischemic stroke patients during a 10-year follow-up [9]. However P-wave axis change has not been assessed nor has P-wave vector magnitude in this population, as the P-wave axis normally corresponds to 60 degrees with similar variability in the frontal plane to the QRS axis with more variability in the transverse and sagittal planes [12]. In regards to voltage assessment, the P-wave terminal force in V_1_ of >0.04 mm/s (PTFV1) has also not reliably been predictive of AF in this same population [8]. Recently, another P-wave time measure, the prolongation of the P-wave duration (Pd) >120 ms along with biphasic morphology in the inferior leads or in aVF and III along with notched p-wave in II, known as advanced inter-atrial block, has been shown to have predictive value for development of atrial fibrillation in ischemic stroke patients [13]. In a 10-year follow-up in ischemic stroke patients, the QRS duration (QRSd) has only had very modest results for predicting AF in ischemic stroke patients [8]. Thus, to date only one useful time-dependent independent 12-lead electrocardiographic predictor for AF in ischemic stroke patients has shown its value (advanced inter-atrial block), whereas no voltage-dependent measures have been tested.

Vectorcardiographic (VCG) principles (3-dimensional parameters, derived from a 12-lead electrocardiogram) have provided additional diagnostic [14, 15] and prognostic [16–20] information, building upon the traditional 12-lead ECG. Dispersion of ventricular depolarization, as measured by the QRS vector magnitude has had predictive value in ventricular arrhythmia development pre-operatively and peri-operatively in patients with congenital heart disease, independent of QRSd [21, 22]. Furthermore, a low P-wave amplitude in lead I is associated with displaced conduction and clinical recurrence of paroxysmal AF post-radiofrequency ablation [23]. A low 3-dimensional P-wave vector magnitude (Pvm), however, has not been assessed in any known cohorts based on the 12-lead ECG or otherwise. Also, this potentially useful tool, which gives the magnitude of the p-wave in 3-dimensional space has yet to be employed for the prediction of AF. Given the relationship between P-wave amplitude and ventricular depolarization duration, further assessment into time-duration and amplitude interrelationship is warranted. To date no relationship of atrial voltage to time duration have been assessed for prediction of AF. Furthermore, P-wave time duration per voltage assessment has therefore also not been assessed in predicting AF in ischemic stroke patients or otherwise.

We aimed to assess the value of the three-dimensional P-wave vector magnitude (Pvm) and its relationship to P-wave duration for prediction of new-onset AF after ischemic stroke.

## Methods

### Study cohort

The original study population originated from the Lund Stroke Register (LSR) and comprised 336 consecutive first-ever ischemic stroke patients included in LSR between March 1, 2001 and February 28, 2002 as it had been described previously [8]. At enrollment in the LSR, 109 ischemic stroke patients had AF detected by ECG screening, medical records review or record linkage with the Swedish National Patient Register as described previously [8] and were excluded from this analysis. All patients enrolled signed written consents. The present study sample therefore comprised of 227 first-ever ischemic stroke patients (median age 73 years at stroke onset (interquartile range 25–75% (IQR 63–80), 92 females) without known AF at inclusion in the LSR. We followed up all study subjects until October 17, 2011, the date when the information from the Swedish National Patient Register was obtained. Informed consent was obtained from all participants included in the LSR. The study was approved by the Lund University Ethics Committee.

### Baseline ECG and clinical assessment

Medical records of all study subjects were analyzed for history of cardiac failure, hypertension, diabetes mellitus, transient ischemic attack (TIA) and ischemic heart disease at baseline. Cardiovascular risk profiles measured by CHADS_2_ and CHA_2_DS_2_-VASc scales [8] were evaluated for the time of inclusion in the LSR in the acute phase when the index ischemic stroke had just occurred.

**Fig. 1:**
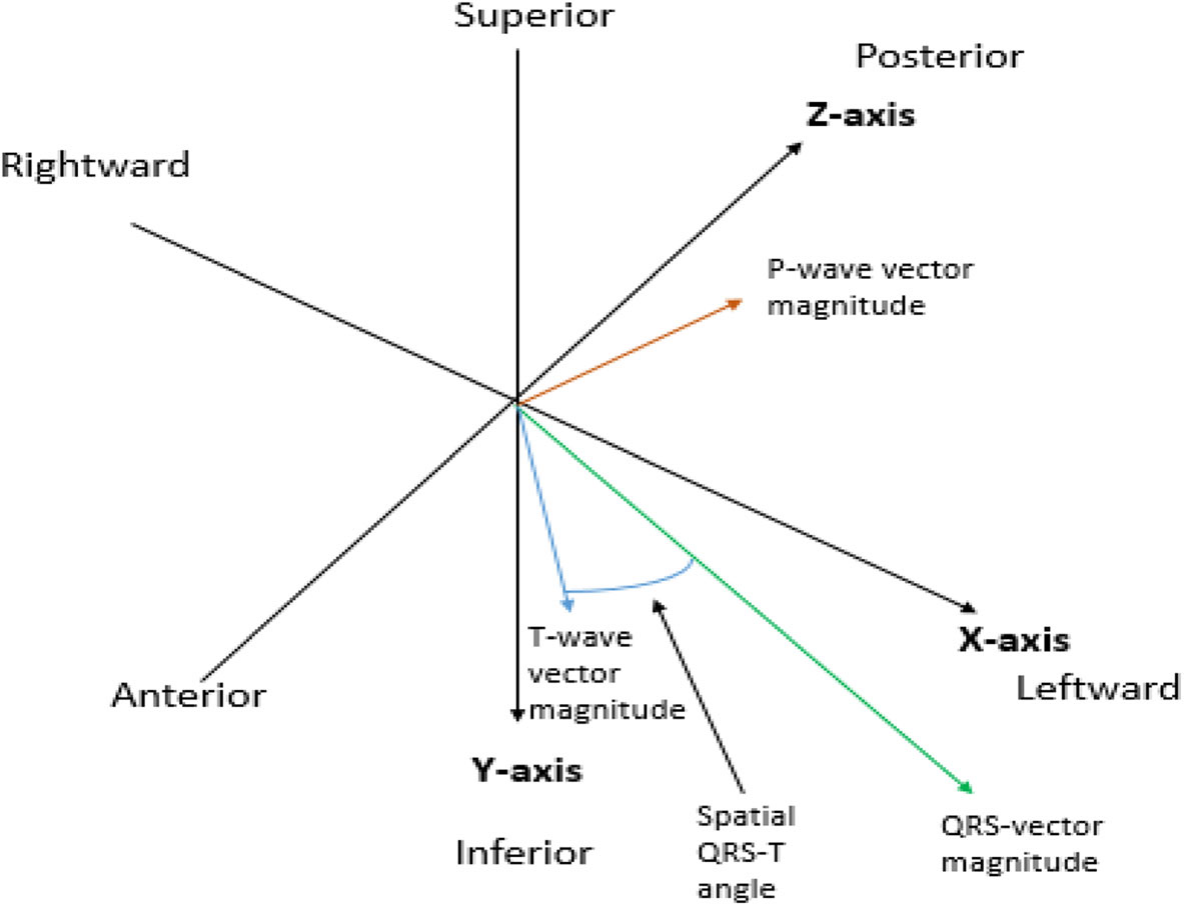
Example of determining the P wave vector magnitude (Pvm) along with QRS and T wave vector magnitudes

**Fig. 2:**
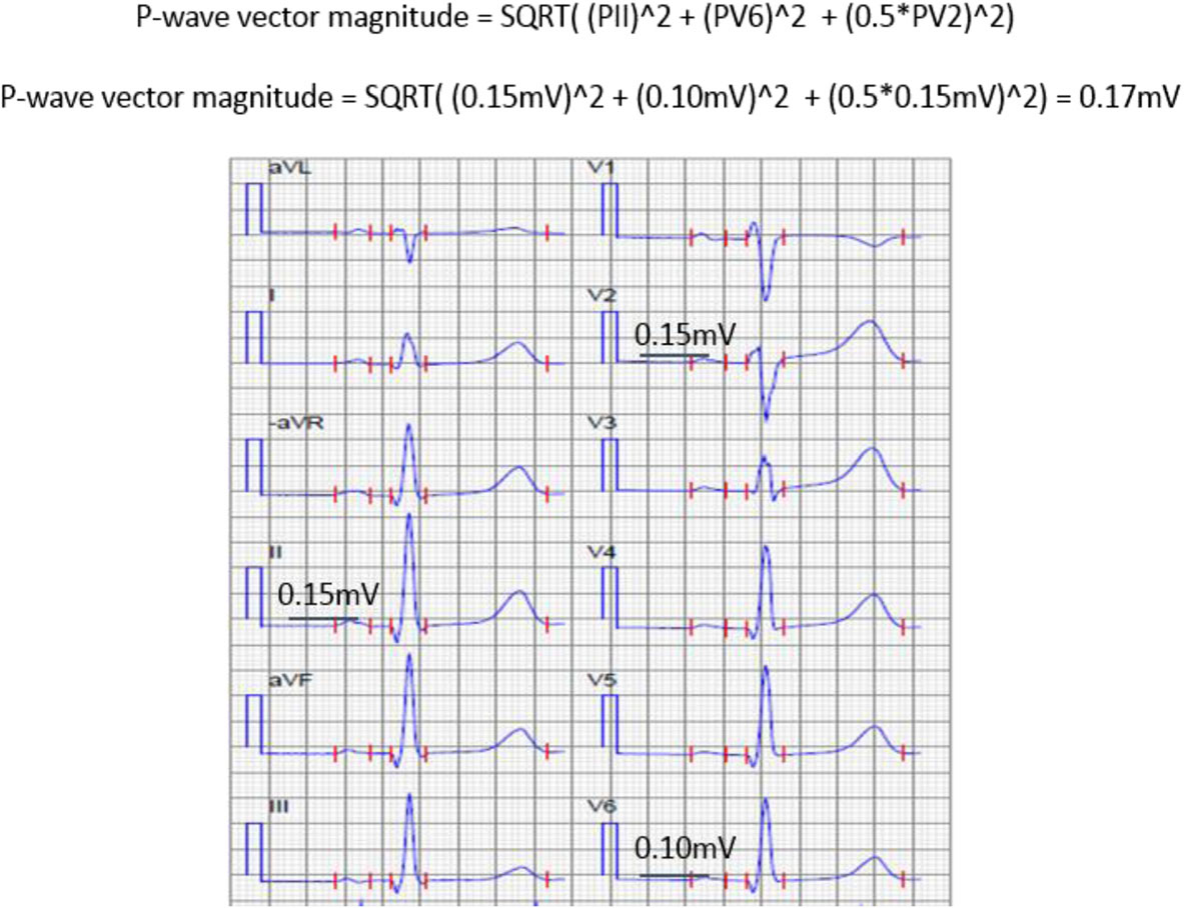
Example of calculation of the P wave vector magnitude (Pvm)

Sinus rhythm ECG recordings obtained at stroke admission with median time from stroke event to ECG registration 0 day (IQR 0–2 days) were extracted from the regional electronic database (GE MUSE, GE Healthcare, MegaCare) and processed offline. The measurements of Pd, QRSd, corrected QT interval (QTc), PQ interval were performed automatically using the University of Glasgow 12-lead ECG analysis algorithm [24]. The Pvm was calculated automatically as the square root of the sum of the squared P-wave magnitudes in leads V6, II and one half of the P-wave amplitude in V2 ($\sqrt{PV{6}^2+{PII}^2+{\left({0.5}^{\ast }PV2\right)}^2}$), based on the P-wave magnitude as defined by the visually transformed Kors’ Quasi-orthogonal method [25, 26]. Please see Figs. 1 and 2. The Pd/Pvm was defined as the Pduration/Pvm and was calculated from the data above automatically utilizing MATLAB R2013b (The MathWorks, Inc., Natick, MA, USA) for Linux.

P wave duration, QRS duration, corrected QT interval and PQ interval were measured in ms. Corrected QT was calculated using Bazett’s formula: QTc = QT/√R-R interval. Pvm was calculated in microvolts. Negative P-wave terminal force in lead V_1_ was also calculated as described previously [8].

### Ascertainment of new-onset AF during follow-up

New onset AF was assessed during the follow-up period starting from the date of enrollment until the end of follow-up or date of death. AF documentation was based on information obtained from the regional electronic ECG archive which contains all ECG recordings taken in the hospital’s local catchment area and also by linkage with national registers: the Swedish Patient Register and the Swedish Causes of Death Register. All available ECG recordings for all study subjects from the date of enrollment until the end of follow-up in 2011 were reviewed for the presence of AF by a trained cardiologist (MB). On surface ECG, AF was defined as a rhythm disorder which lasted sufficiently long for a 12-lead ECG to be recorded, with irregular RR intervals, indistinct P waves and atrial cycle length of 200 ms where distinct atrial activity was visible on surface ECG [27].

The Swedish Patient Register is administered by the Swedish National Board of Health and Welfare and includes data on main and secondary diagnoses at discharge from all public hospitals in Sweden starting in 1987. The register uses International Classification of Disease (ICD) codes with the 10th edition (ICD-10) used from 1997 and until today. The Cause of Death Register is also provided by the Swedish National Board of Health and Welfare and contains information (since 1961) from death records, including underlying causes of death and up to 20 contributory causes of death coded to the current edition (ICD-10). The presence of the ICD-10 code I48 in the Swedish national registers identified AF diagnosis with high specificity and modest sensitivity as we showed recently in a validation study on patients with ischemic stroke enrolled in the LSR [2].

### Statistical methods

Baseline clinical characteristics were compared between stroke patients who developed AF during follow-up and those who remained AF-free using chi-square or Fisher’s exact test for categorical variables and Student’s t-test versus Mann–Whitney U-testing, as appropriate, for continuous variables with an approximately normal distribution or alternatively non-parametric tests, as appropriate. Parametric data are presented as mean ± standard deviation, whereas non-parametric data are presented as median (interquartile range). For log linearity, each variable was categorized into quartiles where applicable and plotted to assess linearity of the quartiles. The primary outcome in this study was defined as occurrence of AF. Subjects who did not develop AF during the 10-year follow-up were censored at time of death or at end of follow-up.

Cox proportional hazard regression models were used to estimate the adjusted hazard ratios (HR) and their 95% confidence intervals (CI) of new onset AF associated with clinical and ECG covariates. Univariate Cox regression analyses were performed separately for each component of CHA_2_DS_2_-VASc score and for each ECG parameter. Clinical factors and ECG parameters significantly associated with new onset AF in the univariate analyses were included in a stepwise regression analysis with backward elimination. Our Cox model was adjusted for known significant clinical covariates (known to predispose to AF or known to have a relationship to the Pd/Pvm). The Kaplan-Meier product-limit method was used to generate a survival curve indicating new onset AF during 10-year follow-up after enrollment in LSR. A Kaplan-Meier curve was also used to demonstrate discernible differences at an optimum cut-off for the Pd/Pvm in identifying incidence of new-onset atrial fibrillation. Optimum cut-off was assessed by the receiver operating characteristic (ROC) curve. Cut-off *p*-values at 0.10 or less were used as entry cut-off values for multivariate analyses. *P* values of 0.05 were considered significant. All analyses were performed using SPSS Statistics 20 (SPSS Inc., Chicago, Illinois, USA). No reproducibility testing was performed given our fully automatic data processing.

## Results

Baseline characteristics of all study subjects at time of enrollment are presented in Table 1. At baseline 227 were fulfilled inclusion criteria and were included in the analysis.

**Table 1:** Baseline clinical characteristics of stroke patients without or with subsequent development of atrial fibrillation

Parameter	Stroke (N=227)	No AF (n=188)	AF (n=39)	*P*-value
Age, years	73 [63 to 80]	73 [61 to 80]	73 [69 to 80]	0.072
Male sex (%)	135 (59%)	114 (61%)	21 (54%)	0.693
Heart Failure	7 (3%)	4 (2.1%)	3 (7.7%)	0.218
Hypertension (%)	130 (57%)	101 (53.7%)	29 (74.4%)	0.012
Diabetes (%)	35 (15%)	26 (13.8%)	9 (23.7%)	0.210
Vascular disease (%)	95 (42%)	77 (41.0%)	18 (46.2%)	0.560
TIA (%)	49 (22%)	45 (23.9%)	4 (10.3%)	<0.001
New-onset atrial fibrillation	39 (17%)	0 (0.0%)	39 (100.0%)	<0.001
Median time to AF onset/end follow-up	3.2 [1.3 to 5.9])	9.7 [4.3 to 10.1]	2.9 [1.2 to 6.4]	<0.001
P duration	116 [106 to 126]	116 [106 to 122]	118 [111 to 131]	0.224
QRSd	78 [68 to 90]	86 [78 to 94]	88 [75 to 99]	0.880
Pvm	0.16 [0.13 to 0.20]	0.16 [0.13 to 0.20]	0.13 [0.11 to 0.19]	0.006
P duration/Pvm	711 [560 to 893]	694 [547 to 862]	801 [586 to 1046]	0.009

Data presented as Median [Interquartile range]

All patients had no evidence of AF in the immediate acute phase after stroke onset

*AF* atrial fibrillation, *TIA* transient ischemic attack, *QRSd* QRS duration, *Pvm* P-wave vector magnitude

### Detection of new onset atrial fibrillation (10-year follow-up)

The median time for follow-up was 9.4 years [IQR 6.1–9.9], 115 (51%) stroke patients died. Complete follow-up data were available for 112 (49%) of the stroke patients. In total, 2588 ECG’s were reviewed with a median number of ECG recordings per person of four (IQR 1–9) [8]. New onset atrial fibrillation was found in 39 (17%) of the stroke patients (Hazard ratio 1.49, 95% confidence interval 0.09–2.35, *p* = 0.121) as previously reported [2, 8]. The median time to AF onset was 3.2 (IQR 1.3 to 5.9) years.

### ECG and clinical predictors of new onset atrial fibrillation after ischemic stroke

On ECGs obtained in the acute phase after stroke onset, the median QRSd was 96 ms (IQR 88–108), the median duration of the P wave was 116 ms (IQR 106–124), and the median PQ interval was 169 ms (IQR 152–188). The median Pvm was 0.15 mV (IQR 0.13 to 0.20) and the median Pd/Pvm was 737 ms/mV (IQR 581 to 955).

Table 2 depicts univariate and multivariate predictors of new-onset atrial fibrillation in stroke patients. Significant univariate predictors of new-onset atrial fibrillation included age > 65 years, presence of hypertension, heart failure, QRSd, and Pd/Pvm (Table 2). No standard ECG characteristics including P-wave duration, QRS duration or negative P-wave terminal force in lead V1 or QRSd were significantly associated with new-onset AF during follow-up. Independent predictors of new-onset atrial fibrillation were instead Pvm/Pd and those parameters considered a moderator of Pvm/Pd including age > 65 years, hypertension, and heart failure (Table 2) [28]. The C-statistic for the model was 0.71 (95% CI 0.61 to 0.82).

**Table 2:** Clinical electrocardiographic predictors of new-onset atrial fibrillation during 10-year follow-up of ischemic stroke patients without known atrial fibrillation at their index stroke

Univariate Cox regression analysis			Multivariate Cox regression analysis	
Parameter	Hazard ratio (95% CI)	*P*-value	Hazard ratio (95% CI)	*P*-value
Age > 65 years	2.88 (1.20 to 6.89)	0.018	1.04 (1.02 to 1.07)	0.001
Hypertension	3.45 (1.40 to 3.49)	0.007	3.21 (1.35 to 7.67)	0.008
Heart failure	4.04 (1.24–13.18)	0.020	2.72 (1.08 to 6.83)	0.033
Diabetes	1.83 (0.87 to 3.87)	0.111		
Male gender	1.22 (0.50 to 1.59)	0.459		
Stroke group	1.391 (0.855 to 2.263)	0.184		
P duration	1.02 (0.96 to 1.05)	0.105		
QRS duration	1.02 (1.00 to 1.04)	0.025	1.01 (1.00 to 1.02)	0.354
PQ interval	1.00 (0.99 to 1.01)	0.966		
Pvm	1.001 (0.994 to 1.009)	0.751		
P duration/Pvm	2.320 (1.367 to 3.938)	0.002	2.02 (1.18 to 3.46)	0.010
P terminal force V1	1.00 (95% CI 1.00–1.00)	0.142		

*Pvm* p-wave vector magnitude, *95% CI* 95% confidence interval

**Fig. 3:**
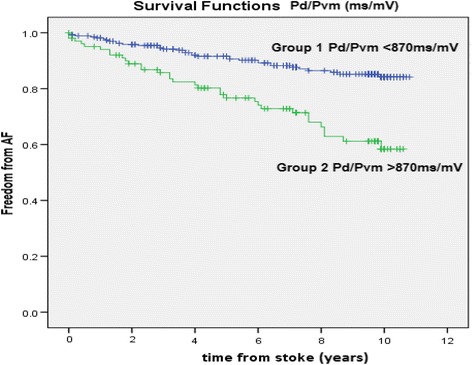
Kaplan-Meijer survival curve from stroke onset to atrial fibrillation detection for p wave duration/p-wave vector magnitude (Pd/Pvm) at a cut-off of 870 milliseconds/millivolt (ms/mV), log rank *p*-value <0.001

The area under the ROC curve value for the Pd/Pvm was 0.63 (0.55 to 0.71, *p* = 0.013). At an optimal cut-off value of 870 ms/mV the sensitivity, specificity, positive and negative predictive values were 51, 79, 40 and 89%, respectively (optimized for highest negative predictive value given the ECG the screening value of the ECG). A Kaplan-Meier curve based on this cut-off value (Fig. 3) provided a *p*-value of <0.001 for differentiation between survival curves for the risk of development AF during 10-year follow-up after first-ever ischemic stroke. Sub-analyses of patients who do not meet any of the independent predictors (ie. without hypertension, who were less than 65 years of age, did not have heart failure and had a Pd/Pvm of less than 870 ms/mV), did had a 93.2% chance of not developing atrial fibrillation. The positive predictive value for development of AF was 27.9% in a patient without hypertension, who were less than 65 years of age and did not have heart failure, but with a Pd/Pvm of less than 870 ms/mV.

## Discussion

Our study demonstrates that a measure of P-wave dispersion by duration and voltage assessment from the standard 12-lead ECG, taken during hospital admission for ischemic stroke, in the form of Pd/Pvm can predict new-onset AF during follow-up while neither P-wave duration nor P-wave terminal force in lead V_1_ were significantly associated with subsequent AF occurrence. The relation of Pd/Pvm to new-onset AF remained independent after adjustment for other clinical parameters.

### Presence of AF

As previously reported, by the end of the 10-year follow-up AF was detected in 15% of our initially AF-free subjects, which corresponds to the reported AF incidence for an aging population of 18% in those older than 85 years by the end of a 7-year follow-up and 17% of those 65–74 years by the end of a 6-year follow-up [9, 29]. Compared with studies based on only ECG screening, studies that employed implantable devices generally have shown higher AF detection rates - of up to 28–30% in patients with ischemic stroke or TIA, as well as in those with risk factors for ischemic stroke [30–32].

### Clinical parameters

We previously reported that in the same cohort age > 65 years, presence of hypertension or heart failure showed a univariate as well as multivariate predictive relation to subsequent AF onset during a 10-year follow-up period [8]. Clinical parameters we found significant in previous analyses were used in the Cox modeling in the current analysis [8, 28].

### P-wave duration divided by 3-dimensional p-wave vector magnitude (Pd/Pvm)

Previous literature mostly has focused on P-wave duration as a predictive tool for new-onset atrial fibrillation with some success in non-ischemic stroke populations [10, 11]. Although in the subgroup of those <60 years of age, the overall P-wave duration yielded a non-significant HR (1.15, 95% CI 0.90 to 1.47). It has also been shown that maximum P wave duration at the upper fifth percentile was associated with long-term AF risk in an elderly community-based cohort [11]. In hypertensive patients the P-wave duration independently predicted the development of new-onset atrial fibrillation [11]. However, in a recent publication of our cohort, no significant predictive value was found for the P-wave duration as a predictor for new-onset AF [8]. In another study, prolonged P-wave duration and advanced interatrial block in particular have shown an association with AF [13]. Furthermore, PTFV1 was not found to be a reproducibly significant predictor of AF [9]. In patients with recurrent atrial fibrillation after catheter ablation, low voltage in lead I (<0.01 mV) was associated with recurrence of AF [21]. Our results demonstrate that atrial voltage dispersion, as measured by Pvm is not in itself alone associated with the development of AF. However, our study is the first to show that atrial time duration versus voltage dispersion (Pd/Pvm) is a potentially clinically useful predictive measure which can be obtained by the ECG alone. Pvm has also shown predictive value for atrial arrhythmias in patients with congenital heart disease, and in particular tetralogy of Fallot patients undergoing pulmonary valve replacements [28]. In this same study Pvm inversely correlated with higher right atrial pressure, left and right ventricular ejection fractions, QRSd, and older age [28]. In the above publication Pvm was predictive of organized right atrial arrhythmias (intra-atrial re-entrant tachycardia and typical flutter), thus in the more disorganized left atrial arrhythmia (AF), it appears time dispersion across the left atrium must also be taken into account. A risk score based on the above parameters might be helpful in ruling out those at risk for atrial fibrillation, and furthermore can be automatically calculated, however further reproducibility with other ECG systems and automated methods would be required. Our study also demonstrated a high specificity and negative predictive value for identification of AF in ischemic stroke patients but with low sensitivity and positive predictive values. This demonstrates that in our cohort although those who develop AF cannot necessarily all be identified (sensitivity 51%) and the value of a Pd/Pvm > 870 ms/mV does not necessarily identify all of those who may develop AF (positive predictive value 30%). Those who do not develop AF, however, are going to be those who have a Pd/Pvm <870 ms/mV. Thus if effectively reproduced independently, this may be a reasonable and cost-effective screening test for those at risk for developing AF.

### Limitations

This study was retrospective and did not use a pre-specified AF screening protocol, thus the number of ECG’s available during follow-up analysis was lower in subjects without detected AF. This may represent an underestimation of AF in patients with asymptomatic AF, given their lack of need to contact health care providers. Also, the ECG search utilized in this study was limited to Southern Sweden’s Skania region, thus other ECG’s possibly performed outside of the Skania region were unavailable for review. Therefore, if a patient was mobile and sought healthcare elsewhere, these ECG’s would not be included. Other prolonged data monitoring such as via implantable devices (e.g. loop recorders) were not available for data analysis. Our data was, however obtained via linkage with the Swedish Patient Register, which for each specific treatment occasion contains up to 20 contributory diagnoses per patient, suggesting that if observed, AF would have been registered in the Patient register. Also, during a 10-year follow-up after stroke a number of confounders such as inability to detect all episodes of AF and lack of reported events such as transient ischemic attacks or other parameters in the CHA_2_DS_2_-VASc score calculation as well as degree of heart failure, which may not have been taken into account. This is also inherently a limitation of Registry data along with lack of atrial size/volume data, which have been shown to be predictors of atrial fibrillation [32, 33]. Furthermore, the Cox model assumes constant effect over time, which may not be complete accurate for every single parameter. Our results need to be viewed in light of these possible confounders. Also the analysis was a post-hoc analysis performed on a prospectively this prospectively enrolled cohort, thus limitations exist regarding assessment of Pd/Pvm compared to other parameters.

## Conclusion

Atrial time dispersion over voltage magnitude, as measured by the Pd/Pvm, appears to have some usefulness in risk stratifying stroke patients for risk of subsequent AF in the post-hoc analysis of an observational study on ischemic stroke survivors. It was the only ECG parameter measured which predicted new-onset AF independently from the clinical covariates. Further prospective studies in larger cohorts, including investigation regarding non-invasive imaging parameter directly compared, are needed to validate its clinical usefulness, however, Pd/Pvm may be worth further investigation for potential usefulness as a relatively simple and easily available clinical tool for AF prediction after ischemic stroke.

## Abbreviations

AF: Atrial fibrillation
ECG: Electrocardiogram
ICD: Internal cardiac defibrillator
LSR: Lund Stroke Register
ms: millisecond
mV: millivolt
Pd: P-wave duration (milliseconds)
PTFV1: P-wave terminal force in V_1_ of >0.04 mm/s
Pvm: P-wave vector magnitude (millivolts)
QRSd: QRS duration
ROC: Receiver operating characteristic curve
TIA: Transient ischemic attack
